# Effectiveness of Dry Needling versus Manual Therapy in Myofascial Temporomandibular Disorders: A Single-Blind Randomized Controlled Trial

**DOI:** 10.3390/jpm13091415

**Published:** 2023-09-21

**Authors:** Rocío García-de la-Banda-García, Irene Cortés-Pérez, María del Rocío Ibancos-Losada, María del Carmen López-Ruiz, Esteban Obrero-Gaitán, María Catalina Osuna-Pérez

**Affiliations:** 1 FISIDEC University Center, University of Córdoba, C/José Aumente Baena s/n, 14940 Cabra, Spain; gbandag@hotmail.com; 2 Department of Health Sciences, Faculty of Health Sciences, University of Jaén, Campus Las Lagunillas s/n, 23071 Jaén, Spain; icortes@ujaen.es (I.C.-P.); mril0001@red.ujaen.es (M.d.R.I.-L.); mlruiz@ujaen.es (M.d.C.L.-R.);

**Keywords:** temporomandibular disorders, dry needling, manual therapy, pain, pressure–pain threshold, trigger points

## Abstract

Dry needling (DN) is an invasive physiotherapy technique employed for reducing myofascial pain. To compare the effectiveness of dry needling (DN) versus manual therapy (MT) in improving pain, active maximal mouth opening (AMMO) and cervical disability in patients with myofascial pain from temporomandibular disorders (TMDs) were investigated against these treatments. A single-blind, randomized controlled trial was carried out. Individuals (*n* = 50) with TMDs were randomly allocated in a 1:1 ratio to the DN (*n* = 25) or MT group (*n* = 25). Each group received three sessions, separated by 4 days, of either DN or MT. Outcomes were assessed according to pain intensity (Numeric Pain Rating Scale), AMMO (cm), disability (Neck Disability Index), and pressure–pain threshold (PPT) (digital algometry) from the active myofascial trigger points. In both groups, pain and neck disability were significantly lower at the end of treatment compared with those measured at baseline (pain: −2.52 with 95% CI: −3.43 to −1.60 for DN group; pain: −2.92 with 95% CI: −3.77 to −2.07 for MT group; disability: −3.2 with 95% CI: −4.31 to −2.09 for DN group; disability: −2.68 with 95% CI: −3.56 to −1.79 for MT group), but not were not lower after the first session, without differences between the groups. AMMO was significantly higher after the first session (0.16 with 95% CI: 0.03 to 0.29 for DN group; 0.30 with 95% CI: 0.20 to 0.41 for MT group) and at the end of treatment in both groups (0.27 with 95% CI: 0.14 to 0.41 for DN group; 0.37 with 95% CI: 0.22 to 0.52 for MT group) compared with the baseline measurements. Finally, PPT results for the masseter and pterygoid muscles were significantly higher at the end of treatment in both groups (without statistically significant differences between groups), but not after the first session. The assessed therapies, DN and MT, are equally effective in improving pain, AMMO, cervical disability, and PPT in the muscles directly involved in the temporomandibular joint biomechanics of patients with myofascial TMDs.

## 1. Introduction

The temporomandibular joint (TMJ) is shaped like a bicondylar diarthrosis, showing a double connection, with the temporal bone and the mandible, and is one of the most complex joints in the human body [1]. Temporomandibular disorders (TMDs) are a heterogeneous set of dysfunctions that present with craniofacial pain, alterations in the masticatory muscles, and temperature changes in the temporomandibular joint and surrounding areas [2,3], affecting orofacial functions such as chewing and speech [4]. TMDs are considered the second leading cause of non-dental pain in the head and orofacial structures, affecting 31% of the adult population worldwide and occurring more frequently in the female population and in young men (20–40 years), but decreasing in prevalence with age [5,6].

The etiology of TMD is considered multifactorial, and includes genetic and physical predisposition, psycho-emotional factors, neck disorders, trauma, and daily habits such as chewing gum or clenching teeth [7]. These types of disorders are usually accompanied by other episodes, such as tension headaches, depression, or anxiety [8]. TMDs are categorized as either intra-articular (within the joint) or extra-articular (involving the surrounding musculature). Musculoskeletal conditions are the most common cause of TMDs, accounting for at least 50% of cases [4]. The pain caused by TMD is able to radiate to other orofacial areas, the neck and the head, causing alterations such as ear pain, headaches, or hyperalgesia in the surrounding muscles [9]. This widespread pain condition is associated with pathophysiological changes in pain processing, mainly the central sensitization phenomenon [10], which is defined as the “increased responsiveness of nociceptive neurons in the central nervous system to their normal or subthreshold afferent input” by the International Association for the Study of Pain (IASP).

In 2013, the International Research Diagnostic Criteria for Temporomandibular Dysfunction Consortium Network published an updated classification structure for TMDs. The most commonly associated syndromes include myofascial pain disorder, disk derangement disorders, osteoarthritis, and autoimmune disorders [11,12]. Myofascial pain disorder must be suspected in patients with pain in the masticatory muscles, along with the existence of painful trigger points on palpation, and limited mouth opening [13]. One of the masticatory muscles most frequently affected is the lateral pterygoid muscle [14,15]. Patients with TMDs often present with a reduced range of motion, deviations in mouth opening, and joint noise. All of these symptoms cause a decrease in their quality of life, limiting the performance of daily tasks and possibly increasing work absenteeism [16].

Currently, there are multiple therapeutic approaches to reduce the symptoms associated with TMDs. The most conservative approaches include pharmacotherapy, dental splints, and traditional physiotherapy; these treatments are minimally aggressive and achieve positive clinical effects. Firstly, pharmacotherapy is indicated to reduce pain and inflammation in the area; however, the indicated substances pose significant risks for patients, such as gastrointestinal or kidney problems [17], if taken continuously for more than four weeks. Secondly, splints are an affordable, minimally invasive method used to correct and align the joint, and they are prescribed mainly in patients with bruxism; however, there is great controversy over their effectiveness [18]. On the other hand, traditional physiotherapy includes a series of passive and active manipulative techniques that seek to reduce pain and increase orofacial function. These techniques include kinesitherapy, stretching, mandibular opening and closing re-education, neuromuscular techniques, electrotherapy, and therapeutic physical exercises, among others. Previous studies report the effectiveness of physiotherapy techniques, compared to pharmacotherapy or splints, in reducing pain and improving temporomandibular function in patients with TMDs [19,20]. However, it is sometimes difficult to manually access the affected area, hence the need for a more invasive approach.

Among the more invasive physiotherapy techniques, dry needling stands out, and is considered minimally invasive. The application of dry needling in myofascial trigger points consists of introducing a low-caliber needle, without additional substances, into the active trigger points to produce a deactivation [21]. There are two ways to perform the technique, depending on the depth reached with the needle: superficial dry needling, where the needle is introduced only to the subcutaneous tissue that covers the trigger point, and the deep technique, in which the needle penetrates the muscle [22]. This technique causes controlled muscle microspasms in the punctured area, after which muscle relaxation occurs. When performing this technique, an analgesic effect is produced as a consequence of its somatosensory involvement, producing relief from local and referred pain [23]. In recent studies, dry needling has shown positive effects in the treatment of pain in some myofascial structures that are difficult to access through manual palpation [15].

The aim of this study was to compare the effectiveness of manual therapy versus dry needling therapy in terms of perceived pain, mouth opening movement, the degree of cervical disability, and the pressure–pain threshold (PPT) of active myofascial trigger points (MTrPs) in patients with temporomandibular joint disorders.

## 2. Materials and Methods

### 2.1. Design and Ethical Requirements

This single-blind randomized controlled trial was registered at clinicaltrials.gov (NCT04469088) and was conducted according to the recommendations of the Consolidated Standards of Reporting Trials (CONSORT) statement for randomized controlled trials by the World Health Organization and the International Committee of Medical Journal Editors (ICMJE). Ethical approval was obtained from the Ethical Committee of the University of Jaén (JUN.20/4.PRY), and the study was conducted according to the Declaration of Helsinki. The randomized controlled trial was carried out between June 2021 and March 2022.

### 2.2. Subjects

Participants between 18 and 65 years old with TMDs were recruited from the Miophys Physiotherapy and Rehabilitation Clinic (Córdoba, Spain) using convenience sampling. Participants had to meet at least two of the following inclusion criteria: (1) pain on palpation of the MTrP of the right or left masseter, lateral pterygoid, and sternocleidomastoid muscles (minimum 3 active trigger points); (2) pain in the temporomandibular joint; (3) limited mouth opening; and (4) clicking of the temporomandibular joint. We considered the following exclusion criteria: fibromyalgia; orthodontia; systemic disease, syndrome, or pathology with possible joint repercussions (including bruxism); patients undergoing treatment with anti-inflammatory drugs; jaw fracture; mandibular surgery; non-collaborative patients; and patients with phobias of needles. After the inclusion criteria were applied, all participants were classified according to original Research Diagnostic Criteria for Temporomandibular Disorders (RDCs/TMDs) [12].

All participants provided written informed consent before enrollment. At the beginning of the study, they were randomly allocated into two intervention groups (dry needling or manual therapy) using the random allocation functions of the Epidat software version 3.1 (Conselleria de Sanidade, Xunta de Galicia, Galicia, Spain).

### 2.3. Interventions

Subjects received 3 sessions of dry needling or manual therapy, separated by 4 days. An expert physiotherapist carried out the interventions in both groups.

#### 2.3.1. Dry Needling Intervention

This treatment consisted of puncturing the possible active MTrP of the right or left masseter, lateral pterygoid, and sternocleidomastoid muscles. MTrPs are defined as hypersensitive spots within taut bands of skeletal muscle, which are painful upon compression and trigger characteristic referred pain. MTrPs are classified as active when they cause spontaneous pain and latent when they only provoke pain when stimulated.

Patients in this group received dry needling in at least 1 active trigger point and in a maximum of 6. Sterile needles, 0.26 mm in diameter by 40 mm in length, guided with a plastic cannula of the brand Ener-qi, were used. The area was cleaned with alcohol and deep puncturing of the myofascial trigger point was performed, triggering local spasm responses. The needle was moved up and down through the muscle following the technique described by Hong [24]. After the procedure, the area was compressed with cotton for 90 s.

#### 2.3.2. Manual Therapy Intervention

**Figure 1:**
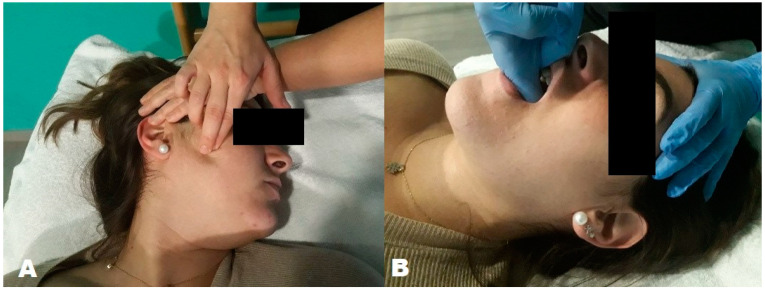
Neuromuscular technique on right masseter muscle (**A**) and Jones technique in left lateral pterygoid muscle (**B**).

This treatment consisted of the application of the neuromuscular technique to the right and left masseter (Figure 1A) and sternocleidomastoid muscles, and the Jones technique or ischemic compression to the right and left lateral pterygoid muscles (Figure 1B). Ischemic compression to the masticatory muscles is effective in relieving pain and increasing maximum mouth opening [25]. The neuromuscular technique was performed with the index and middle fingers to apply transverse friction with enough pressure to feel the vertical fibers of each muscle or produce a medium discomfort level. To perform the Jones technique on the right and left lateral pterygoid muscles, the patient was asked to open their mouth and deviate their jaw slightly towards the treated side in order to make room for the finger to be located between the upper jaw and the coronoid process. Once the trigger point had been located, pressure was applied in order to manage for pain, followed by jaw movements aimed at achieving neurological silence. Pressure was maintained for 90 s. After this period, pressure was slowly removed, and the initial position was passively recovered.

### 2.4. Variables and Time Assessment

Sociodemographic information (age and gender) was collected from each patient. The main study variables were pain, active maximal mouth opening (AMMO), cervical disability, and PPT of possible active MTrPs (all muscles were explored on both sides: right and left masseter, right and left lateral pterygoids, and right and left sternocleidomastoids). All variables, with the exception of disability, were assessed at baseline before treatment (T0 assessment), one hour after the first session (T1 assessment) to study the immediate effect, and two weeks after completing the treatment (T2 assessment) so that possible post-session effects could be avoided. Assessments were performed by a physiotherapist with more than 10 years of experience in the field and different from the one who performed the interventions.

The Numeric Pain Rating Scale (NPRS) was used to assess the intensity of temporomandibular pain in patients [26]. The NPRS is a scale divided at 1-point intervals, on which patients can mark the intensity of their temporomandibular pain from 0 (“no pain”) to 10 (“worst possible pain imaginable”).

The range of maximal mouth opening was assessed by measuring the distance (in cm) between the maxillary and mandibular incisal edges of the incisor teeth, using a validated ruler in centimeters [27].

Neck disability was measured using the Spanish version of the Neck Disability Index (NDI) questionnaire [28]. The NDI is a self-report questionnaire used to determine how neck pain affects a patient’s daily life. Each of the 10 items is scored by the patient from 0 to 5. The maximum score is therefore 50. Higher scores indicate worse outcomes. This scale presents high internal consistency (Cronbach alpha: 0.89) and a large intraclass correlation (0.98) [28].

The PPT in each active MTrP was measured with pressure algometry using the Wagner Force Ten™ FDX digital algometer (www.wagnerinstruments.com, accessed on 5 December 2022). This algometer is a portable, low-cost digital force gauge, and it is commercially available as a mechanical nociceptive threshold testing device that has been validated for use in various species [29]. A numerical value was provided for the subjective perception of pain in each muscle tested. The unit of measurement used was kg/cm^2^. Once the active MTrP was located within the taut band, pressure was exerted perpendicular to the muscle plane and on the active myofascial trigger points through the support surface of the device. The pressure was increased by 1 kg per second. When the pressure became painful, the individual raised their hand to warn us.

### 2.5. Statistical Analysis

Statistical analyses were performed using the statistical package SPSS 21.00 (SPSS Inc., Chicago, IL, USA) by a blinded statistical physiotherapist. Mean and standard deviation were included in the data description in continuous variables, and frequencies and percentages in categorical variables. To study the homogeneity of the groups at the start of the intervention, we used the one-way ANOVA for quantitative variables and the Chi-squared test for categorical variables. For the differences between groups in terms of pain intensity, maximal mouth opening, and PPT in each muscle, a 2-by-3 mixed ANOVA was conducted with time (baseline, post-first-session, and post-treatment) as the within-subject factor and group (dry needling and manual therapy) as the between-subject factor. For the differences between groups in neck disability, a 2-by-2 mixed model ANOVA was conducted with time (baseline and post-treatment) as the within-subject factor and group as the between-subject factor. Post hoc pairwise comparisons were analyzed using the Student’s *t*-test with Bonferroni correction. The partial eta squared (ηp^2^) was used as an estimator of the size of the main effects and interactions between the ANOVAs, for which a value between 0.2 and 0.49 indicated a small effect size, a value between 0.5 and 0.79 a medium effect size, and a value > 0.8 indicated a large effect [30]. Significance was determined at an α level of 0.05, and a 95% confidence interval (CI) was assumed for each analysis.

## 3. Results

**Figure 2:**
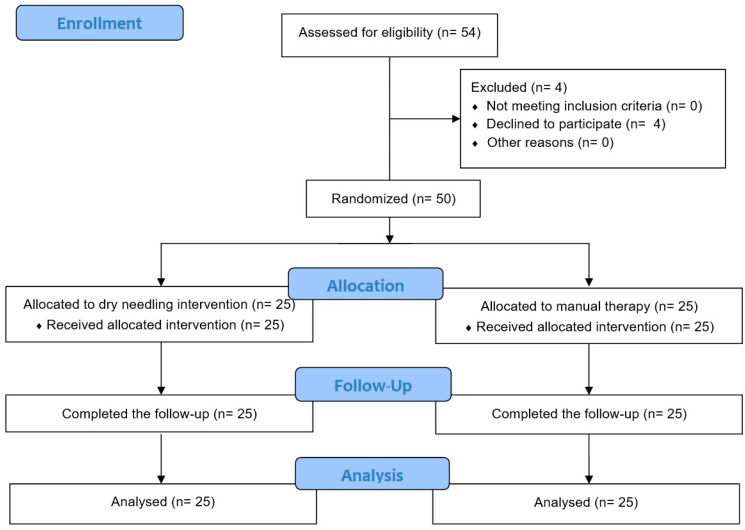
CONSORT flow diagram.

The final sample was composed of 50 participants who met the inclusion criteria (Figure 2). Twenty-five patients comprised the DNG, with a mean age of 34.56 ± 8.43 years old (12 males and 13 females). The other 25 patients comprised the manual therapy group (MTG) with a mean age of 38.08 ± 9.75 years old (9 males and 16 females). All patients included were diagnosed in the Axis I of RDCs/TMDs (*n* = 36 with myofascial pain and *n* = 14 with myofascial pain and limited opening). There were no statistically significant differences between the two groups at baseline in terms of age, gender, or outcome variables. Table 1 shows the baseline and outcome variables of patients in both the DNG and MTG.

### 3.1. Pain Intensity

**Figure 3:**
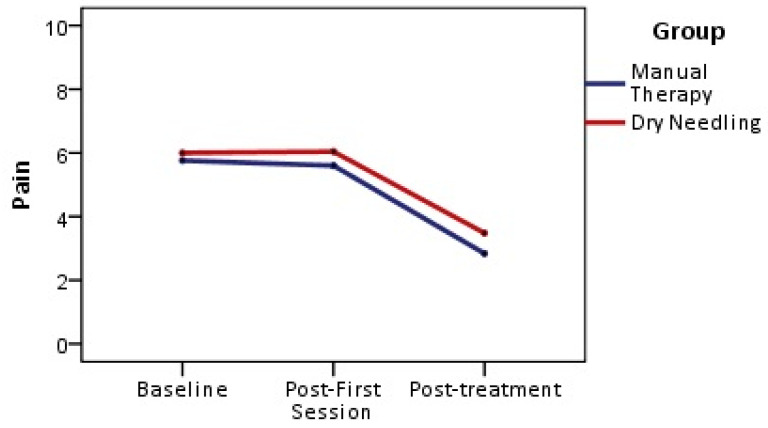
Numeric pain rating scale. Differences within and between groups.

The 2-by-3 mixed model ANOVA for pain intensity revealed a significant main effect for time (F = 80.68, *p* < 0.001; ηp^2^ = 0.627), with a medium effect size value, but with no significant difference between groups (F = 1.136; *p* = 0.292; ηp^2^ = 0.023). There was a non-significant time-by-group interaction (F = 0.436; *p* = 0.512; ηp^2^ = 0.009). Post hoc comparisons are presented in Table 2. There was a reduction in pain for the general study sample, but without significant differences between groups. More specifically, pain was significantly lower at the end of treatment compared with that at the baseline, but not after the first session (Figure 3).

### 3.2. Active Maximal Mouth Opening

**Figure 4:**
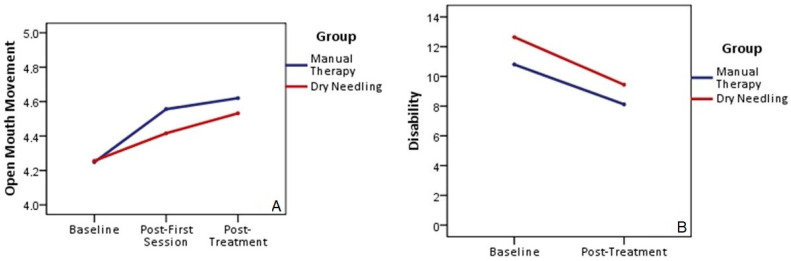
Maximum mouth opening (**A**) and cervical disability (**B**). Differences within and between groups.

The 2-by-3 mixed model ANOVA for active maximal mouth opening revealed a significant main effect for time (F = 43.468, *p* < 0.001; ηp^2^ = 0.475), close to a medium effect size value, but with no significant differences between groups (F = 0.38; *p* = 0.541; ηp^2^ = 0.008). There was a non-significant time-by-group interaction (F = 0.954; *p* = 0.334; ηp^2^ = 0.019). The post hoc comparisons are presented in Table 2. There was an increase in mouth opening movement in the overall study sample, but no differences between groups were observed. Open mouth movement was significantly higher post-first-session and at the end of treatment compared with that at the baseline (Figure 4).

### 3.3. Cervical Disability

The 2-by-2 mixed model ANOVA for cervical disability revealed a significant main effect for time (F = 73.079, *p* < 0.001; ηp^2^ = 0.606), with a medium effect size value, but with no significant differences between groups (F = 1.918; *p* = 0.172; ηp^2^ = 0.038). There was a non-significant time-by-group interaction (F = 0.576; *p* = 0.451; ηp^2^ = 0.012). Post hoc comparisons are presented in Table 2. Disability was significantly lower at the end of treatment compared with that at the baseline in both groups (Figure 4).

### 3.4. Pressure–Pain Threshold of the Left and Right Masseters, Lateral Pterygoids, and Sternocleidomastoids

**Figure 5:**
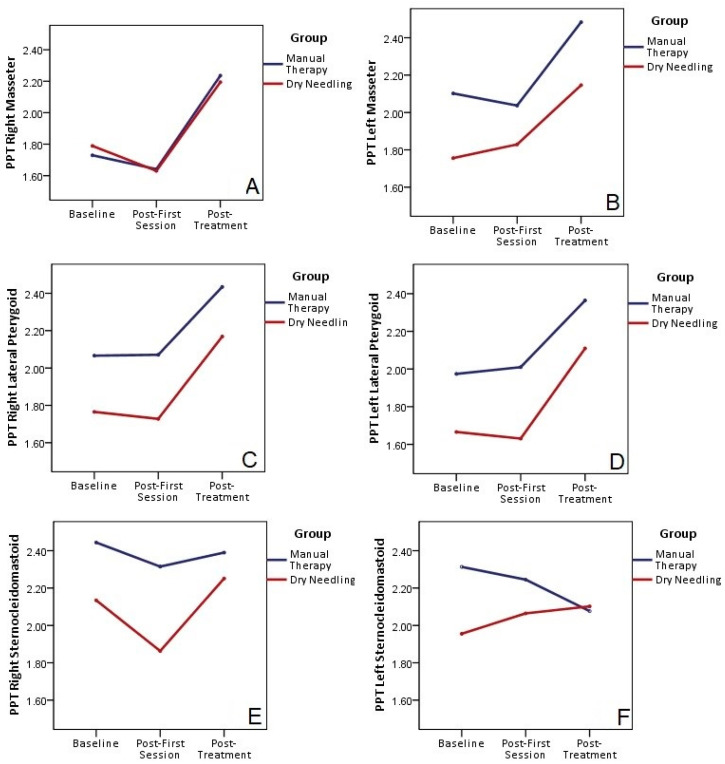
Pressure–pain threshold for right and left masseter (**A**,**B**), lateral pterygoid (**C**,**D**) and sternocleidomastoid muscles (**E**,**F**). Differences within and between groups.

Table 3 and Figure 5 show the PPT assessment in the right and left masseter, lateral pterygoid, and sternocleidomastoid muscles. The repeated measures ANOVA model test (two groups × three times) for PPT in the right masseter showed a significant main effect for time (F = 15.830, *p* < 0.001; ηp^2^ = 0.361), with a small effect size value, but with no significant effect for group (F = 0.001; *p* = 0.993; ηp^2^ = 0.001). There was a non-significant time-by-group interaction (F = 0.196; *p* = 0.661; ηp^2^ = 0.007). There was an increase in the pressure–pain threshold for this muscle, but without differences between groups. More specifically, the PPT results were significantly higher at the end of treatment compared with those at the baseline, but not after the first session.

The repeated measures ANOVA model test (two groups × three times) for PPT in the left masseter showed a significant main effect for time (F = 24.395, *p* < 0.001; ηp^2^ = 0.411), with a small effect size value, but no significant effect for group (F = 1.530; *p* = 0.224; ηp^2^ = 0.042). There was a non-significant time-by-group interaction (F = 0.004; *p* = 0.952; ηp^2^ = 0.001). PPT results for this muscle were significantly higher at the end of the treatment compared with those at the baseline, but not after the first session, and without differences between groups.

The repeated measures ANOVA model test (two groups × three times) for PPT in the right lateral pterygoid showed a significant main effect for time (F = 19.208, *p* < 0.001; ηp^2^ = 0.434), with a small effect size value, but no significant effect for group (F = 1.947; *p* = 0.175; ηp^2^ = 0.072). There was a non-significant time-by-group interaction (F = 0.040; *p* = 0.844; ηp^2^ = 0.002). There was an increase in the pressure–pain threshold for this muscle, but without differences between groups. More specifically, the PPT results were significantly higher at the end of treatment compared with those at the baseline, but not after the first session.

The repeated measures ANOVA model test (two groups × three times) for PPT in the left lateral pterygoid showed a significant main effect for time (F = 4.561; *p* = 0.044; ηp^2^ = 0.172), but no significant effect for group (F = 1.671; *p* = 0.209; ηp^2^ = 0.071). There was a non-significant time-by-group interaction (F = 0.019; *p* = 0.892; ηp^2^ = 0.001). The PPT results for this muscle were significantly higher at the end of treatment compared with those at the baseline, but not after the first session.

The repeated measures ANOVA model test (two groups × three times) for PPT in the right sternocleidomastoid showed no significant main effect for time (F = 0.140, *p* = 0.711; ηp^2^ = 0.006) in either group (F = 1.499; *p* = 0.233; ηp^2^ = 0.061). There was a non-significant time-by-group interaction (F = 1.044; *p* = 0.318; ηp^2^ = 0.043).

The repeated measures ANOVA model test (two groups × three times) for PPT in the left sternocleidomastoid showed a non-significant main effect for time (F = 0.096, *p* = 0.760; ηp^2^ = 0.004) in both groups (F = 0.506; *p* = 0.483; ηp^2^ = 0.020). There was a non-significant time-by-group interaction (F = 1.734; *p* = 0.200; ηp^2^ = 0.065).

## 4. Discussion

The results of this study indicate that, after completing the full treatment in each group (three sessions), dry needling and manual therapy both proved effective in reducing pain and associated cervical disability, as well as improving active mouth opening ROM and increasing PPT in the active MTrPs of masseters and lateral pterygoids muscles.

Previous reviews have shown that both the classical physiotherapy approach and dry needling can be effective in reducing the signs and symptoms of TMDs [12,27,28]. However, the paucity of studies directly comparing the efficacy of these two approaches made it necessary to carry out this study.

Regarding the evaluation of pain intensity (assessed with the NPRS), none of the groups showed a post-session reduction (T1 evaluation). However, at the end of the full treatment (T2 assessment), there were clinically relevant (medium effect) and statistically significant reductions in pain in both groups, although no statistically significant differences were found between the groups. These results present great clinical relevance, since both therapies are effective in minimizing pain intensity, exceeding the minimum clinically important difference (MCID) value for the NPRS in patients with TMDs (between 1.5 and 3.2 points) [31]. Thus, dry needling achieved a mean pain reduction of −2.52 points, and manual therapy achieved a mean of −2.92 points, with each thus showing a medium effect size. On the one hand, the results of the effect of dry needling in our study coincide with those of the study by Dib-Zakkour et al. (2022), in which a significant reduction in facial pain was found after the dry needling of active MTrPs [32]. Furthermore, Özden et al. (2018) reported that dry needling was effective in reducing pain in patients with temporomandibular pain, although these authors reported that superficial dry needling (needle in the direction of the active MTrP, penetrating at least 10 mm) may be better than deep dry needling of the masseter muscles (entering the active MTrP masseter) in reducing pain [33]. One of the reasons why pain did not improve after the first session in either group may be related to the possible adverse effects of this therapy—mainly, the residual pain after the application of the dry needling technique.

We assessed the effects of both therapies on the PPT of the right and left masseter, lateral pterygoid, and sternocleidomastoid muscles, with the following findings at the end of the therapy: After the full treatment, both dry needling and manual therapy demonstrated effectiveness in reducing pain sensitivity, although no effect was reported on the sternocleidomastoid muscles. Our findings did not show statistically significant differences between therapies after the full treatment, so we can suggest that both therapies, separately, are effective. These findings show that dry needling is an effective therapy for reducing the mechanosensitivity of pain in the masseter and lateral pterygoid muscles, after the end of the intervention. Our findings regarding the effect of dry needling in the masseter muscle are in line with previous studies. Fernández-Carnero et al. (2010) reported that the practice of dry needling in the active masseter MTrP reduces mechanosensitivity to pain in patients with TMDs [34]. Blasco-Bonora et al. (2017) reported that dry needling in the masseter and active temporalis MTrP reduces pain and improves PPT [35]. Finally, Özden et al. (2018) showed that the superficial dry needling of the masseter may be better than deep dry needling in improving PPT [33]. Two previous reviews have shown that dry needling may be effective for improving PPT in patients with TMDs, although these findings present a very low quality of evidence [36,37]. As such, the findings of our study and others recently published, such as those by Dib-Zakkour et al. (2022), can provide robustness to the meta-analyses that were performed [32].

We found that both dry needling and manual therapy are effective in increasing AMMO in patients with TMDs after the first session and after the interventions have finished, although no statistically significant differences between therapies were established. Previous investigations have reported that the MCID in active mouth opening varies between 6.5 and 9 mm [38]. Neither of these therapies exceeded these MCID values, although our findings may be underestimated due to the small sample size. In addition, our findings show that both therapies are effective for increasing the AMMO in these patients, and represent two physiotherapy-based approaches to the masticatory and neck muscles that may be used in the management of patients with TMDs and mouth opening ROM restrictions. Our findings are in line with the recent RCT of Dunning et al. (2022), who reported that dry needling shows better outcomes than conservative techniques, such as occlusal splints and joint mobilization, in reducing pain intensity and increasing AMMO right after finishing treatment [39]. However, it is important to clarify that, in that study, dry needling was used in combination with upper cervical manipulation.

Finally, it is important to remark that patients with TMDs present clinical signs and symptoms of cervical disability or dysfunction [40]. Pain in musculoskeletal cervical structures may manifest in jaw muscles due to the connection and convergence between craniofacial and cervical afferents in the upper cervical nociceptive neurons and trigeminocervical nucleus [41]. The greatest reduction in the magnitude of pain in the temporomandibular joint zone may explain the reported improvements in cervical disability assessed with NDI after the therapy. Our findings showed that both therapies are effective in reducing cervical disability after the full treatment (−3.2 for DNG and −2.68 points for MTG), without differences between therapies. However, although these therapies are effective for reducing cervical disability, they did not exceed the MCID value for NDI (3.5 points) [42,43]. It is possible that the improvements in cervical disability are also related to reductions in the mechanosensitivity to pain in the muscles directly involved in temporomandibular biomechanics, improving the functionality of the joint and the well-being of the surrounding anatomical areas and reporting; therefore, an improvement in cervical disability was observed. It would be very interesting to follow the example of Dunning et al. (2022), who combined the dry needling of masticatory muscles with a cervical intervention (cervical spinal manipulation) in patients with TMDs, and obtained better results, probably due to this trigeminocervical connection [39].

The current study has several limitations. Firstly, the lack of blinding for participants and assessors may lead to an overestimation of the therapeutic effects of both interventions, reducing the accuracy of treatment effects, due to potential performance and detection biases. Secondly, the influence of the placebo effect and/or the natural evolution of TMD on our results cannot be determined. Thirdly, there was a lack of follow-up. Only one measurement was made at the end of both interventions (immediate effect, 2 weeks later), so the medium-, and long-term effects of these therapies have not been reported. In the future, it would be essential to carry out a similar study while seeking to reduce these limitations, so as to increase the generalizability and precision of the results.

Findings present in this study are clinically relevant for clinical practice, due to showing that both therapies are effective in the management of patients with TMDs, allowing the physiotherapist to choose between invasive therapies, such as dry needling, or classical therapies, such as manual therapy, according to the preference of patients. In the future, it would be advisable to carry out more research, increasing the number of patients per group and the follow-up time after treatment.

## 5. Conclusions

Dry needling and manual therapy are equally effective in the management of patients with TMDs, as the findings demonstrate comparable post-session and post-treatment results. At the end of treatment, both therapies showed significant reductions in pain and cervical disability, with medium effect size values; an improvement in active mouth opening, with effect size values close to medium; and an increase in Pressure–Pain Threshold in the active myofascial trigger points of masseters and lateral pterygoid muscles, with a small effect size value.

## Figures and Tables

**Table 1:** Baseline demographic and outcome variable characteristics of the patients included in the study.

Variables		Dry Needling Group (*n* = 25)	Manual Therapy Group (*n* = 25)	*p*
Age (years)		34.56 ± 8.43	38.08 ± 9.75	0.127
Gender	Male, *n* (%)	12 (48)	9 (36)	0.39
	Female, *n* (%)	13 (52)	16 (64)	
NPRS (0–10)		6 ± 1.63	5.76 ± 1.36	0.587
MMO (cm)		4.25 ± 0.46	4.24 ± 0.58	0.435
NDI (0–50)		12.64 ± 5.03	10.8 ± 4.66	0.622
PPT (kg/cm^2^)	Right Masseter	1.78 ± 0.87	1.72 ± 0.57	0.101
	Left Masseter	1.75 ± 0.8	2.1 ± 0.7	0.414
	Right Lateral Pterygoid	1.76 ± 0.8	2.14 ± 0.58	0.226
	Left Lateral Pterygoid	1.66 ± 0.64	1.97 ± 0.79	0.504
	Right Sternocleidomastoid	2.13 ± 0.68	2.44 ± 0.6	0.749
	Left Sternocleidomastoid	1.95 ± 0.58	2.31 ± 0.8	0.187

Abbreviations: *n*, number of participants; NPRS, Numeric Pain Rating Scale; MMO, maximal mouth opening; NDI; Neck Disability Index; PPT, pressure–pain threshold. Note: Data are given as mean ± standard deviation; *p* < 0.05: significant difference.

**Table 2:** Differences within groups in pain, active maximal mouth opening, and cervical disability.

Variable	Point Assessment	Dry Needling Group (*n* = 25)	Manual Therapy Group (*n* = 25)
NPRS (0–10)	Baseline	6.00 ± 1.63	5.76 ± 1.36
	Post-First-Session	6.04 ± 2.42	5.60 ± 1.78
	Within-Group Differences from Baseline	0.40 (−0.90, 0.98)	−0.16 (−0.83, 0.51)
	Post-Treatment	3.48 ± 2.00	2.84 ± 1.95
	Within-Group Differences from Baseline	−2.52 (−3.43, −1.60) *	−2.92 (−3.77, −2.07) *
MMO (cm)	Baseline	4.25 ± 0.46	4.24 ± 0.58
	Post-First-Session	4.41 ± 0.38	4.55 ± 0.50
	Within-Group Differences from Baseline	0.16 (0.03, 0.29) *	0.30 (0.20, 0.41) *
	Post-Treatment	4.53 ± 0.31	4.62 ± 0.46
	Within-Group Differences from Baseline	0.27 (0.14, 0.41) *	0.37 (0.22, 0.52) *
NDI (0–50)	Baseline	12.64 ± 5.03	10.80 ± 4.66
	Post-Treatment	9.44 ± 3.08	8.12 ± 3.79
	Within-Group Differences from Baseline	−3.2 (−4.31, −2.09) *	−2.68 (−3.56, −1.79) *

Abbreviations: *n*, number of participants; NPRS, Numeric Pain Rating Scale; MMO, maximal mouth opening; NDI; Neck Disability Index. Note: Data are given as mean ± standard deviation, including within-group mean difference, with 95% confidence interval; *p* < 0.05: significant difference *.

**Table 3:** Differences within groups in pressure–pain threshold in active MTrP of masseters, lateral pterygoids, and sternocleidomastoids.

	Dry Needling Group	Manual Therapy Group
**PPT Right Masseter (*n* = 30)**	***n* = 16**	***n* = 14**
Baseline	1.78 ± 0.87	1.72 ± 0.57
Post-First-Session	1.63 ± 0.91	1.64 ± 0.52
Within-Group Differences from Baseline	−0.15 (−0.43, 0.11)	−0.08 (−0.31, 0.14)
Post-Treatment	2.19 ± 0.98	2.23 ± 0.58
Within-Group Differences from Baseline	0.40 (0.04, 0.76) *	0.50 (0.18, 0.83) *
**PPT Left Masseter (*n* = 37)**	***n* = 23**	***n* = 14**
Baseline	1.75 ± 0.80	2.10 ± 0.70
Post-First-Session	1.82 ± 0.74	2.03 ± 0.63
Within-Group Differences from Baseline	0.07 (−0.08, 0.23)	−0.06 (−039, 0.26)
Post-Treatment	2.14 ± 0.80	2.48 ± 0.84
Within-Group Differences from Baseline	0.39 (0.20, 0.58) *	0.38 (0.09, 0.66) *
**PPT Right Lateral Pterygoid (*n* = 27)**	***n* = 14**	***n* = 13**
Baseline	1.76 ± 0.80	2.06 ± 0.50
Post-First-Session	1.72 ± 0.65	2.07 ± 0.44
Within-Group Differences from Baseline	−0.3 (−0.34, 0.27)	0.004 (−0.18, 0.19)
Post-Treatment	2.16 ± 0.72	2.43 ± 0.49
Within-Group Differences from Baseline	0.40 (0.07, 0.73) *	0.36 (0.19, 0.34) *
**PPT Left Lateral Pterygoid (*n* = 32)**	***n* = 14**	***n* = 18**
Baseline	1.66 ± 0.67	1.97 ± 0.79
Post-First-Session	1.63 ± 0.59	2.01 ± 0.80
Within-Group Differences from Baseline	−0.04 (−0.24, 0.16)	0.01 (−0.19, 0.22)
Post-Treatment	2.10 ± 0.71	2.36 ± 0.84
Within-Group Differences from Baseline	0.50 (0.29, 0.71) *	0.53 (0.25, 0.80) *
**PPT Right Sternocleidomastoid (*n* = 25)**	***n* = 13**	***n* = 12**
Baseline	2.13 ± 0.68	2.44 ± 0.60
Post-First-Session	1.86 ± 0.74	2.31 ± 0.53
Within-Group Differences from Baseline	−0.27 (−0.57, 0.02)	−0.12 (−0.26, 0.007)
Post-Treatment	2.25 ± 0.71	2.39 ± 0.62
Within-Group Differences from Baseline	0.11 (−0.11, 0.34)	−0.05 (−0.34, 0.23)
**PPT Left Sternocleidomastoid (*n* = 27)**	***n* = 15**	***n* = 12**
Baseline	1.95 ± 0.58	2.31 ± 0.80
Post-First-Session	2.06 ± 0.75	2.24 ± 0.88
Within-Group Differences from Baseline	0.10 (−0.22, 0.43)	−0.06 (−0.33, 0.19)
Post-Treatment	2.10 ± 0.69	2.07 ± 0.76
Within-Group Differences from Baseline	0.14 (−0.21, 0.50)	−0.23 (−0.78, 0.31)

Abbreviations: *n*, active myofascial trigger points; PPT, pressure–pain threshold. Note: Data are given as mean ± standard deviation, including within-group mean difference, with 95% confidence interval; *p* < 0.05: significant difference *.

## Data Availability

Data are available on request due to privacy/ethical restrictions.
